# Implementing mentor mothers in family practice to support abused mothers: Study protocol

**DOI:** 10.1186/1471-2296-12-113

**Published:** 2011-10-18

**Authors:** Maartje JW Loeffen, Sylvie H Lo Fo Wong, Fred PJF Wester, Miranda GH Laurant, Antoine LM Lagro-Janssen

**Affiliations:** 1 Radboud University Nijmegen Medical Centre, Department of Primary and Community Care, Gender & Women's Health, Postbox 9101, 6500 HB Nijmegen, The Netherlands; 2 Radboud University Nijmegen, Faculty Social Sciences, Section Methods and Techniques, Postbox 9104, 6500 HE Nijmegen, The Netherlands; 3 Radboud University Nijmegen Medical Centre, Scientific Institute for Quality of Healthcare, Postbox 9101, 6500 HB Nijmegen, The Netherlands

## Abstract

**Background:**

Intimate partner violence is highly prevalent and mostly affects women with negative consequences for their physical and mental health. Children often witness the violence which has negative consequences for their well-being too. Care offered by family physicians is often rejected because abused women experience a too high threshold. Mentor mother support, a low threshold intervention for abused mothers in family practice, proved to be feasible and effective in Rotterdam, the Netherlands. The primary aim of this study is to investigate which factors facilitate or hinder the implementation of mentor mother support in family practice. Besides we evaluate the effect of mentor mother support in a different region.

**Methods/Design:**

An observational study with pre- and posttests will be performed. Mothers with home living children or pregnant women who are victims of intimate partner violence will be offered mentor mother support by the participating family physicians. The implementation process evaluation consists of focus groups, interviews and questionnaires. In the effect evaluation intimate partner violence, the general health of the abused mother, the mother-child relationship, social support, and acceptance of professional help will be measured twice (t = 0 and t = 6 months) by questionnaires, reporting forms, medical records and interviews with the abused mothers. Qualitative coding will be used to analyze the data from the reporting forms, medical records, focus groups, interviews, and questionnaires. Quantitative data will be analyzed with descriptive statistics, chi square test and t-test matched pairs.

**Discussion:**

While other intervention studies only evaluate the feasibility and effectiveness of the intervention, our primary aim is to evaluate the implementation process and thereby investigate which factors facilitate or hinder implementation of mentor mother support in family practice.

## Background

Domestic violence is highly prevalent and mostly affects women and children [1]. It has negative consequences for the physical and mental health of the victim. Poor mental health, as depression and anxiety, is the largest contribution to the burden of disease associated with intimate partner violence [2]. Children often witness the mental and/or physical abuse of their mother which has negative consequences for their well-being too. Children living with intimate partner violence are at increased risk of developing emotional and behavioral problems [3]. These children run the same risk to develop a depression or substance abuse or commit suicide as those children who were abused themselves [4]. They also are increased at risk to become a victim or perpetrator [4,5]. Therefore witnessing intimate partner violence as a child is considered as child abuse. Early identification of the violence is important to reduce these harmful effects for women and their children. Although family physicians often develop a long trusting relationship with their patient, they often do not recognize the violence [6]. After identification of intimate partner violence, it is important to offer adequate support. A communicative approach with empathy or empowering is valued most by these women [7]. The support that's currently offered by family physicians is often not suited for a woman's stage of change resulting in rejection of care offered by family physicians. However, there are two effective interventions that can overcome these problems.

The first effective intervention is training of family physicians in the identification and discussion of violence. Lo Fo Wong et. al demonstrated that an educational training of family physicians improves detection and discussion of the violence [8]. The second intervention is the introduction of trained mentor mothers in family practice. They can improve the acceptance of professional help by mother and child. It is based on the fact that social support is associated with good physical and mental health outcomes for women [9]. This intervention is primarily developed in Australia and showed an improvement of important clinical outcomes, as reduction of violence and improvement of general well-being [10,11]. The mentor mother support intervention has been modified to the Dutch situation by the department of Gender & Women's Health, Radboud University Nijmegen Medical Centre. The training for mentor mothers was further developed focusing on empowerment regarding four main themes: 1) safety, 2) social support, 3) depressive symptoms, and 4) children witnessing intimate partner violence. Feasibility and effectiveness of this intervention has been studied in Rotterdam: the MeMoSA (Mentor Mothers for Support and Advise) project 2007-2010. In the MeMoSA project Rotterdam mentor mothers were introduced in family practice and preliminary results showed a decrease in violence, a decrease in mental health problems, an increase in acceptance of professional help for mother and child, and an increase in social support and activities [unpublished data, Prosman GJ, Lo Fo Wong SH, Lagro-Janssen ALM].

We believe that mentor mother support is an effective approach justified to be implemented in family practice. To optimize the implementation, it is necessary to know the facilitators and barriers of implementation.

## Objectives

### Primary objective

Determine which factors facilitate or hinder implementation of mentor mother support in family practice.

### Secondary objective

Evaluate the effect of mentor mother support in a different region.

## Methods/Design

### Study design

An observational study with pre- en posttests will be performed.

### Participants

#### Family physicians

Hundred fourteen family physicians, located in Nijmegen and surroundings received a training (described later) in recognizing and discussing intimate partner violence between September 2009 and January 2011. All trained family physicians and their colleagues working in the same family practice, have been invited for participation in the mentor mother support study. Non-respondents were approached by telephone within 3 weeks. Finally 86 family physicians out of 33 family practices, with about 110.000 patients, signed up to participate. Participants will document all mothers who are victims of intimate partner violence, meeting the inclusion criteria (described later) and are asked to enroll them. In focus groups several participating family physicians will discuss the factors that facilitate or hinder implementation of mentor mother support in family practice. All family physicians have to fill in a questionnaire at the end of the study.

#### Mentor mothers

Mentor mothers were recruited by healthcare contact persons of an abused women's shelter organization, involved family physicians, and a school for social healthcare. Recruitment and selection was based on the following criteria: 1) at least intermediate vocational education in healthcare, 2) motivated to become a mentor mother, 3) writing and verbal communicative skills, 4) stability, and 5) cultural diversity between mentor mothers. Interested women were asked to apply for this job and send their curriculum vitae. Ten women were invited for an interview and eight women were employed and trained by the mentor coordinator. The mentor mothers received a contract for 18 months for 8 hours a week. At the end of the study all mentor mothers will take part in a focus group.

#### Mentor coordinator

The mentor coordinator was recruited from the network of the local abused women's support and shelter organization. She is educated as social worker with training experience. The mentor coordinator is responsible for the training and supervision of mentor mothers, she matches the abused mothers with mentor mothers, and is the contact person for the participating family physicians.

#### Mothers who are victims of intimate partner violence

**Figure 1:**
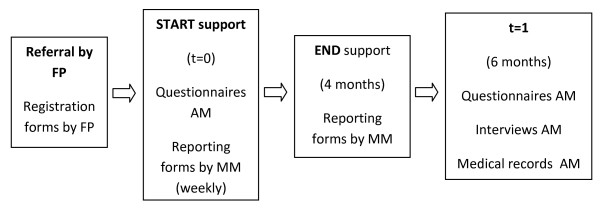
**Data collection effect evaluation**. FP = family physician, MM = mentor mother, AM = abused mother, t = 0: at the start of mentor mother support, t = 1: 6 months after the start of mentor mother support

Family physicians will enroll abused mothers. The inclusion criteria are: 1) detected **or **suspected intimate partner violence **and **2) the woman is a mother of children living at home **or **pregnant. The exclusion criteria are: 1) serious psychiatric problems, as psychosis, which requires psychiatric treatment, 2) serious physical disease which needs hospitalization, or 3) no contact possibilities with the mentor mother at home or at a family practice. Participating mothers have to fill out an informed consent form. In this form she agrees with 1) mentor mother support, 2) filling in a questionnaire twice (at the start of the support (t = 0) and 6 months after the start of the support (t = 1), 3) a personal interview, 6 months after the start of the support, and 4) insight in her medical record. (See figure 1).

### Intervention

#### Mentor mother support

Mentor mother support consists of: one hour weekly visit by a mentor mother, during 4 months, providing non-judgmental active listening and support, developing a trusting relationship and empowering. The aim of the support is to 1) achieve a reduction of the violence, 2) an expansion of social support and an increase of the acceptance of professional help, 3) learn the abused mother to cope with depression/depressive symptoms, and 4) helping abused mothers to become aware of the effect on their child(ren). To lower the threshold for acceptance of help, mentor mother support will be offered as support for mothers who experience difficulties with children living at home and not as a domestic violence support. Cultural background will be taken into account with matching when preferred by the abused mother. Visits will take place at home, at the family practice or any other place where the mother feels safe and comfortable. Every visit will be documented on a form and at the end the family physician will receive a written report.

In order to provide this specific support, mentor mothers received ten days of training previous to the start of mentor mother support. The training has been developed for the Dutch situation in Rotterdam and elaborates on 4 important themes: 1) providing safety and safety behaviors, 2) expansion of social support to break through the isolation of the mother and her child(ren), 3) coping with depressive symptoms and the acceptance of professional help, and 4) the effect of witnessing intimate partner violence on children and the acceptance of help for their child(ren). Mentor mothers and mentor coordinator meet every 2-4 weeks to discuss the content of the coaching and problems mentor mothers encounter. There is also a possibility to receive extra training. For urgent questions the mentor mother can contact the mentor coordinator by phone. All mentor mothers were provided with mobile phones for their personal safety.

#### Training family physicians

To improve recognition of intimate partner violence, family physicians were trained in small groups (12-15 participants) during 3 hours between September 2009 and January 2011. The aim of the training is to enhance awareness of non-obvious signs of intimate partner violence, to increase active questioning and to improve professional attitude, and response to abused women.

### Outcome measures

#### • Primary outcome

The facilitators and barriers of successful implementation of mentor mother support in family practice are the primary outcomes and will be analyzed at four different levels [12]:

■ **Individual**. The individual level focuses on cognitive, motivational and behavioral factors and characteristics of the abused mothers, mentor mothers, mentor coordinator, and family physicians. For example, the motivation of family physicians to pay attention to intimate partner violence might be an important factor that influences implementation.

■ **Social context**. At the social context level professional development, teams and networks of the family physicians, mentor mothers and mentor coordinator will be evaluated. Consensus about the effectiveness of mentor mother support within a family practice for instance, probably facilitates successful implementation.

■ **Organizational**. Analysis on the organizational level focuses on organizational structures, processes and available resources within family practice. For example, limited room availability within a family practice, where mentor mother and abused mother can meet, might hinder successful implementation of mentor mother support.

■ **Society**. Financing, laws and regulation, and characteristics within the group of professionals will be analyzed at the level of society. For example, financing of mentor mother support and paying attention to the problem of intimate partner violence by the government probably facilitates implementation.

Based on the results of mentor mother support in Australia [11] and Rotterdam [unpublished data, Prosman GJ, Lo Fo Wong SH, Lagro-Janssen ALM], we consider implementation of mentor mother support successful when the following 4 goals are accomplished:

1) Identification of at least 65 women who meet the inclusion criteria by participating family physicians.

2) Family physicians offering mentor mother support to at least 55 eligible women.

3) Acceptance of mentor mother support by at least 45 women who are offered mentor mother support.

4) At least 35 women completing mentor mother support.

#### • Secondary outcomes

The secondary outcomes of the study are:

■ Intimate partner violence

■ The general health of the mother

■ The mother-child relationship

■ Social support

■ Acceptance of professional help

With these outcomes we evaluate the effect of mentor mother support implemented in a different region and completely integrated in the family practice.

#### Sample size

We consider a decrease of one third of the total intimate partner violence score 6 months after the start of mentor mother support clinical relevant. To estimate the sample size for the secondary objective of our study that focuses on the effect of mentor mother support, we used the results of the effect evaluation of mentor mother support in Rotterdam [unpublished data, Prosman GJ, Lo Fo Wong SH, Lagro-Janssen ALM]. Because the standard deviations for the mean changes were not available, these data were estimated from the standard deviations for the pre-treatment and post-treatment measurements as recommend by Cochrane Collaboration, using a more conservative correlation coefficient (r = 0.4) [13,14]. With levels of 80% power and 95% confidence this requires recruitment of 14 abused mothers. Based on the results of the the study of mentor mother support in Rotterdam [unpublished data, Prosman GJ, Lo Fo Wong SH, Lagro-Janssen ALM], we estimate a loss of one third of abused mothers accepting mentor mother support. This finally requires recruitment of 21 abused mothers.

### Data collection

#### • Evaluation of the implementation process

Registration forms will be used to investigate if the goals for successful implementation (described above) are accomplished. This means that family physicians have to fill in a registration form for every woman who meets the inclusion criteria. Mentor mothers register if abused mothers complete mentor mother support.

**Figure 2:**
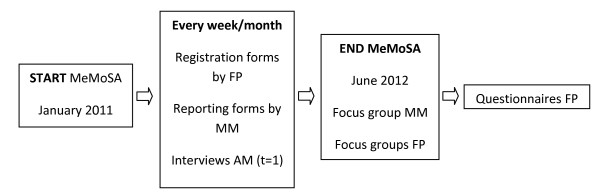
**Data collection implementation process**. FP = family physician, MM = mentor mother, AM = abused mother, t = 1: 6 months after the start of mentor mother support.

Focus groups with several family physicians, who have or have not identified intimate partner violence and have or have not referred to a mentor mother, will be organized. Mentor mothers will also take part in a focus group focused on the implementation process. The list of subjects for the focus groups will be based on the evaluation of mentor mother support in Rotterdam, and on a theoretical framework for possible barriers and facilitators [12]. The results of the focus groups will be used to develop a questionnaire for all participating family physicians. The abused mothers will be interviewed 6 months after the start of mentor mother support (t = 1). (See figure 2).

#### • Effect evaluation

■ ***Intimate partner violence ***will be measured by the Composite Abuse Scale (CAS) [15] at the start (t = 0) and 6 months after the start (t = 1) of mentor mother support. This questionnaire is validated for the measurement of the presence, severity and type of intimate partner violence.

■ The ***general health ***of the mother will be measured by the Symptom Checklist (SCL-90) [16] at the start (t = 0) and 6 months after the start (t = 1) of mentor mother support. This questionnaire measures physical and mental health complaints.

■ The ***mother- child relationship ***will be measured using the NOSIK [17] at the start (t = 0) and 6 months after the start (t = 1) of mentor mother support. This structured questionnaire is the Dutch translation of the Parenting Stress Index Short Form (PSI-SF) [18] and measures the experienced stress of a parent.

■ ***Social support ***will be measured by using reporting forms made by a mentor mother and an interview with the abused mother at the end of the support.

■ ***Acceptance of professional help ***for mother and child(ren) will be measured by using reporting forms filled in by a mentor mother, the medical record and an interview with the abused mother at the end of the support.

### Data analysis

#### • Evaluation of the implementation process

The interviews with the abused mothers, focus group with all mentor mothers, and focus groups with family physicians and their questionnaires will be analyzed focusing on facilitators and barriers of implementation on the four different levels described above. Qualitative data from the interviews and focus groups will be analyzed with qualitative coding in ATLAS.ti. Focus groups and interviews will be recorded and transcribed with participants' consent. Two researchers will study the transcripts independently, identify themes and establish the definite codes. Consensus will be reached in mutual discussion. Subsequently these outcomes will be formulated and interpreted in the research group for final results. Quotes will be used to underline these results. The questionnaires filled in by family physicians will consist of closed and open questions. Closed questions will be analyzed with descriptive statistics in SPSS 16.0, and open questions will be analyzed with qualitative coding in ATLAS.ti.

#### • Effect evaluation

Quantitative data from the questionnaires (CAS, SCL-90, and NOSIK) will be analyzed with descriptive statistics in SPSS 16.0. Associations will be studied with the chi square test. T-test matched pairs will be used to test if the outcome measures (described above) differ significantly between pre- and posttest. Quantitative data from reporting forms and medical records will be analyzed with descriptive statistics in SPSS 16.0. Qualitative data from the reporting forms, medical records, and interviews with the abused mothers, will be analyzed with qualitative coding in ATLAS.ti.

Upon consultation the Medical Ethics Committee of the Radboud University Nijmegen Medical Centre stated that ethical approval was not necessary. (27-06-2008)

## Discussion

The high prevalence and harmful effects of intimate partner violence emphasize the need for adequate help for this vulnerable group of women with children. Mentor mother support developed in Australia and modified to the Dutch situation proved to be feasible and effective. It is unique because it offers semi professional support in family practice which lowers the threshold to acceptance of help. Although there is some overlap between the studies in Rotterdam and Nijmegen, this study has several innovative features. Firstly, our primary aim is to investigate which factors facilitate or hinder a successful implementation of mentor mother support in family practice. Other studies only focused on feasibility and effectiveness and didn't evaluate the implementation process. By knowing the factors that facilitate or hinder implementation we can adjust them and thereby optimize the implementation of mentor mother support in family practice. Secondly we aim to assess whether the effect of mentor mother support is comparable between Rotterdam and Nijmegen, because there are demographic differences between both populations. For example mean income and number of immigrants differ between both regions [19]. Thirdly, it's important to consider the mother-child relationship, because continuing abuse affects the relationship of the mother and her child(ren). It has a negative impact on parenting and on quality of the mother-child bonding. A secure attachment to a non-violent parent seems to be an important protective factor in diminishing trauma and distress [5]. Fourthly, on account of the experiences in Rotterdam adjustments were made that can improve the effect of mentor mother support. This resulted in the idea of offering mentor mother support in the family practice and is expected to lower the threshold for the acceptance of mentor mother support.

So while other studies primarily focused on effect evaluation, this study primarily investigates factors that contribute to the success or failure of the implementation. It offers the opportunity to strengthen the facilitating factors and take away the barriers for implementation and thereby optimizes the implementation of this low threshold intervention for mothers who are victims of intimate partner violence.

## Competing interests

The author declares that they have no competing interests.

## Authors' contributions

SLFW and ALJ are responsible for the design of the study. ML, FW, and MLa participated in the design of the study. ML wrote the first draft of the manuscript and SLFW, FW, MLa, and ALJ revised the manuscript critically. All authors read and approved the final manuscript.

## Authors' information

ML is a psychologist, family physician trainee and PhD student at the department Gender & Women's Health, Primary and Community Care, Radboud University Nijmegen Medical Centre, the Netherlands. SLFW is a family physician and senior researcher at the department Gender & Women's Health, Primary and Community Care, Radboud University Nijmegen Medical Centre, the Netherlands. FW is an academic professor Communication Sciences at the Radboud University Nijmegen, the Netherlands. MLa is a senior research fellow at the Scientific Institute for Quality of Healthcare, Radboud University Nijmegen Medical Centre, the Netherlands. ALJ is a family physician and academic professor Gender & Women's Health at the Radboud University Nijmegen Medical Centre, the Netherlands.

## Pre-publication history

The pre-publication history for this paper can be accessed here:

http://www.biomedcentral.com/1471-2296/12/113/prepub
